# Stable Carbon and Nitrogen Isotopes in a Peat Profile Are Influenced by Early Stage Diagenesis and Changes in Atmospheric CO_2_ and N Deposition

**DOI:** 10.1007/s11270-011-1001-8

**Published:** 2012-01-25

**Authors:** Alice J. Esmeijer-Liu, Wolfram M. Kürschner, André F. Lotter, Jos T. A. Verhoeven, Tomasz Goslar

**Affiliations:** 1 Palaeoecology, Institute of Environmental Biology, Utrecht University, Budapestlaan 4, 3584 CD Utrecht, the Netherlands; 2 Department of Geosciences, University of Oslo, PO Box 1047, Blindern, 0316 Oslo, Norway; 3 Ecology and Biodiversity, Institute of Environmental Biology, Utrecht University, Padualaan 8, 3584 CA Utrecht, the Netherlands; 4 Faculty of Physics, A. Mickiewicz University, Umultowska 85, 61-614 Poznan, Poland; 5 Poznań Radiocarbon Laboratory, ul. Rubież 46, 61-612 Poznań, Poland

**Keywords:** Carbon dioxide, Nitrogen deposition, Stable isotopes, Peat, *Sphagnum fuscum*, Diagenesis

## Abstract

In this study, we test whether the δ^13^C and δ^15^N in a peat profile are, respectively, linked to the recent dilution of atmospheric δ^13^CO_2_ caused by increased fossil fuel combustion and changes in atmospheric δ^15^N deposition. We analysed bulk peat and *Sphagnum fuscum* branch C and N concentrations and bulk peat, *S. fuscum* branch and *Andromeda polifolia* leaf δ^13^C and δ^15^N from a 30-cm hummock-like peat profile from an Aapa mire in northern Finland. Statistically significant correlations were found between the dilution of atmospheric δ^13^CO_2_ and bulk peat δ^13^C, as well as between historically increasing wet N deposition and bulk peat δ^15^N. However, these correlations may be affected by early stage kinetic fractionation during decomposition and possibly other processes. We conclude that bulk peat stable carbon and nitrogen isotope ratios may reflect the dilution of atmospheric δ^13^CO_2_ and the changes in δ^15^N deposition, but probably also reflect the effects of early stage kinetic fractionation during diagenesis. This needs to be taken into account when interpreting palaeodata. There is a need for further studies of δ^15^N profiles in sufficiently old dated cores from sites with different rates of decomposition: These would facilitate more reliable separation of depositional δ^15^N from patterns caused by other processes.

**Electronic supplementary material:**

The online version of this article (doi:10.1007/s11270-011-1001-8) contains supplementary material, which is available to authorized users.

## Introduction

Increased human activity over the past two centuries has resulted in major environmental changes, including an exponential increase in atmospheric carbon dioxide (CO_2_) concentration, from 287 to 369 ppm between 1850 and 2000 (Friedli et al. 1986; Keeling and Whorf 2005), largely caused by a higher rate of fossil fuel combustion (IPCC 2008). Another major environmental change was the dramatic increase in nitrogen (N) emission and deposition in Europe after approximately 1950 (Bleeker and Erisman 1996; de Ruiter et al. 2006; Fowler et al. 2004; Freyer et al. 1996; Holland et al. 1999; Pitcairn et al. 1995; Planbureau voor de Leefomgeving 2008; Thomas et al. 1988). This was followed by a slow decrease after the 1980s in most of Europe due to the implementation of emission-limiting regulations. Although absolute N deposition has decreased, it remains higher than before 1950. Post-1950 N emissions result primarily from fossil fuel burning (predominantly NO_*x*_) and volatisation from intensive agricultural systems (predominantly NH_*y*_) (Asman et al. 1998; Holland et al. 1999). These have led to an increase in wet deposition of nitrate (NO_3_^−^) and ammonium (NH_4_^+^), as well as dry deposition of gaseous nitric acid (HNO_3_), ammonia (NH_3_), NO_*x*_, and particulate NO_3_^−^ (Asman et al. 1998; Erisman et al. 1998; Lawrence et al. 2000).

Changes in atmospheric CO_2_ concentration and N emission and deposition are, respectively, accompanied by variations in the proportions of stable carbon and nitrogen isotopes. Because fossil fuels are composed of ancient organic carbon, which has a much lower ^13^C-to-^12^C isotopic ratio, expressed as δ^13^C, than modern atmospheric CO_2_ (Rigby et al. 1981), the δ^13^C of atmospheric CO_2_ shows a globally decreasing trend from −6.45‰ around 1850 to −8.08‰ in 2002 (Francey et al. 1999; Friedli et al. 1986; Keeling et al. 2005a, b). While the change in atmospheric δ^13^C–CO_2_ is a slow but continuous global decrease, values of δ^15^N in wet and dry deposition show broad spatial variation at all geographic levels, from the global to the local (Bauer et al. 2000; Kelly et al. 2005; Russell et al. 1998). Even at a single location, there may be substantial temporal variation between seasons (Bauer et al. 2000; Freyer 1991; Gao 2002; Russell et al. 1998). This variation is probably caused by the relatively large influence of local sources, such as industry, roads or agriculture. Generally, however, the mean δ^15^N of NH_4_^+^ wet deposition has a more negative value than the δ^15^N of NO_3_^−^ (Bauer et al. 2000; Gao 2002) because of the nature of urea production and the fractionation during volatilization of NH_3_ (Macko and Ostrom 1994; Nadelhoffer and Fry 1994). Also, the mean or median δ^15^N of NH_4_^+^ and/or NO_3_^−^ in wet deposition is most often found to be well below 0‰ (Freyer 1991; Gao 2002; Russell et al. 1998; Xiao and Liu 2002).

N deposition is trapped by plants as both wet and dry deposition and is taken up via foliage, twigs, branches, stems and roots (Harrison et al. 2000). While uptake rates of both NH_*y*_ and NO_*x*_ increase linearly with the deposition concentration, generally NH_*y*_ is taken up faster and in larger amounts than NO_*x*_, typically via a wet surface (Harrison et al. 2000). After uptake, N is transported to sites where it is converted into amino acids and proteins (Harrison et al. 2000). During most of these steps, fractionation against ^15^N is possible (Hobbie and Ouimette 2009). However, such fractionation may decline when N is not abundant.

Many palaeoecological studies use peat as a natural environmental archive. For instance, temperature and bog surface wetness have been inferred by analyses of humification, pollen, plant macrofossils or testate amoebae from peat (Barber et al. 2003; Hendon et al. 2001; Langdon et al. 2003; Mauquoy and Barber 2002). Variations in peat δ^13^C that are related either to mechanisms of CO_2_ uptake by *Sphagnum* or the carbon composition of peat are used for palaeohydrology and climatic reconstructions (Jones et al. 2010; Loisel et al. 2010; Menot and Burns 2001; Nichols et al. 2009; Skrzypek et al. 2009). Variations in peat δ^15^N, related to differences in N sources, N acquisition strategies, vegetation and species shifts, decomposition and hydrology (hummock versus hollow), are used as an indicator of past nutrient status and are also used to make palaeohydrological and climatic reconstruction (Asada et al. 2005; Jones et al. 2010; Wooller et al. 2003).

Due to the lack of stomata in bryophytes, the transfer of atmospheric CO_2_ to the intercellular spaces should be less influenced by environmental conditions such as temperature and light (White et al. 1994). However, there are a variety of factors that need to be taken into account in such interpretations, such as the uptake of CO_2_ that is released during the decomposition of underlying peat layers (Price et al. 1997; Smolders et al. 2001), decomposition or diagenesis itself (Agren et al. 1996; Bostrom et al. 2007; Fernandez and Cadisch 2003), possible CH_4_ fixation (Raghoebarsing et al. 2005), respiration, microclimate (Loisel et al. 2009; Price et al. 1997), sample selection (Moschen et al. 2009) and changing atmospheric δ^13^C (Penuelas and Azcon-Bieto 1992; Zhao et al. 2001). In particular, the influence of the last factor has rarely been investigated in peat studies. At the same time, there is evidence that the N isotope signature of vegetation, particularly bryophytes and specifically *Sphagnum*, reflects the isotopic signature of the N source (Bragazza et al. 2005; Jung et al. 1997; Pearson et al. 2000; Penuelas and Filella 2001; Saurer et al. 2004; Solga et al. 2005; Stewart et al. 2002). In the case of *Sphagnum* spp., this may be related to the positive, non-linear relationship between increased N availability or deposition and bryophyte tissue N concentration (Berendse et al. 2001; Lamers et al. 2000; Limpens and Berendse 2003a; Pitcairn et al. 1995; Tomassen et al. 2003).

As N deposition across Europe varies temporally and spatially (Harrison et al. 2000), the δ^15^N of peat and its peat-forming mosses (*Sphagnum* spp.) can potentially be used as natural archives of past nitrogen deposition or possibly the ratio between NO_*x*_ and NH_*y*_. However, then problems associated with the large natural variability in isotopic values in both N types need to be overcome. In this study, we tested if stable carbon and nitrogen isotopes can be used in natural peat archives to trace changes in atmospheric CO_2_ and N deposition through time.

Our study focused on an ombrogenous peatland in Finland, in northern Scandinavia. While the chance of capturing the atmospheric carbon dioxide concentration is more or less the same for all peat-accumulating ecosystems, ombrogenous peatlands possess the highest potential as biomonitors for atmospheric N input. These are hydrologically isolated from minerotrophic groundwater, receiving their water and N only via atmospheric deposition or N fixation (Cleveland et al. 1999; Opelt et al. 2007; van Breemen 1995). Furthermore, compared to central Europe, northern Scandinavia has a low atmospheric N deposition level, although it has undergone the same variation in N deposition as the central European countries. These factors and the local, relatively cold climate make peat systems from this region very suitable for studying atmospheric N deposition through time. Expecting a shift in isotope signatures of organic matter in the same direction as the shift in its atmospheric precursors, we hypothesised that both the δ^13^C and δ^15^N of pre-anthropogenic periods (i.e. younger than 1850 for C and younger than 1950 for N) will be higher than that of post-anthropogenic periods. Subsequently, we studied whether the vertical gradients in δ^13^C and δ^15^N in this profile can, respectively, be linked to the recent dilution of atmospheric CO_2_ with ancient C and to increased atmospheric anthropogenic N deposition.

## Material and Methods

### Site and Sampling

**Fig. 1:**
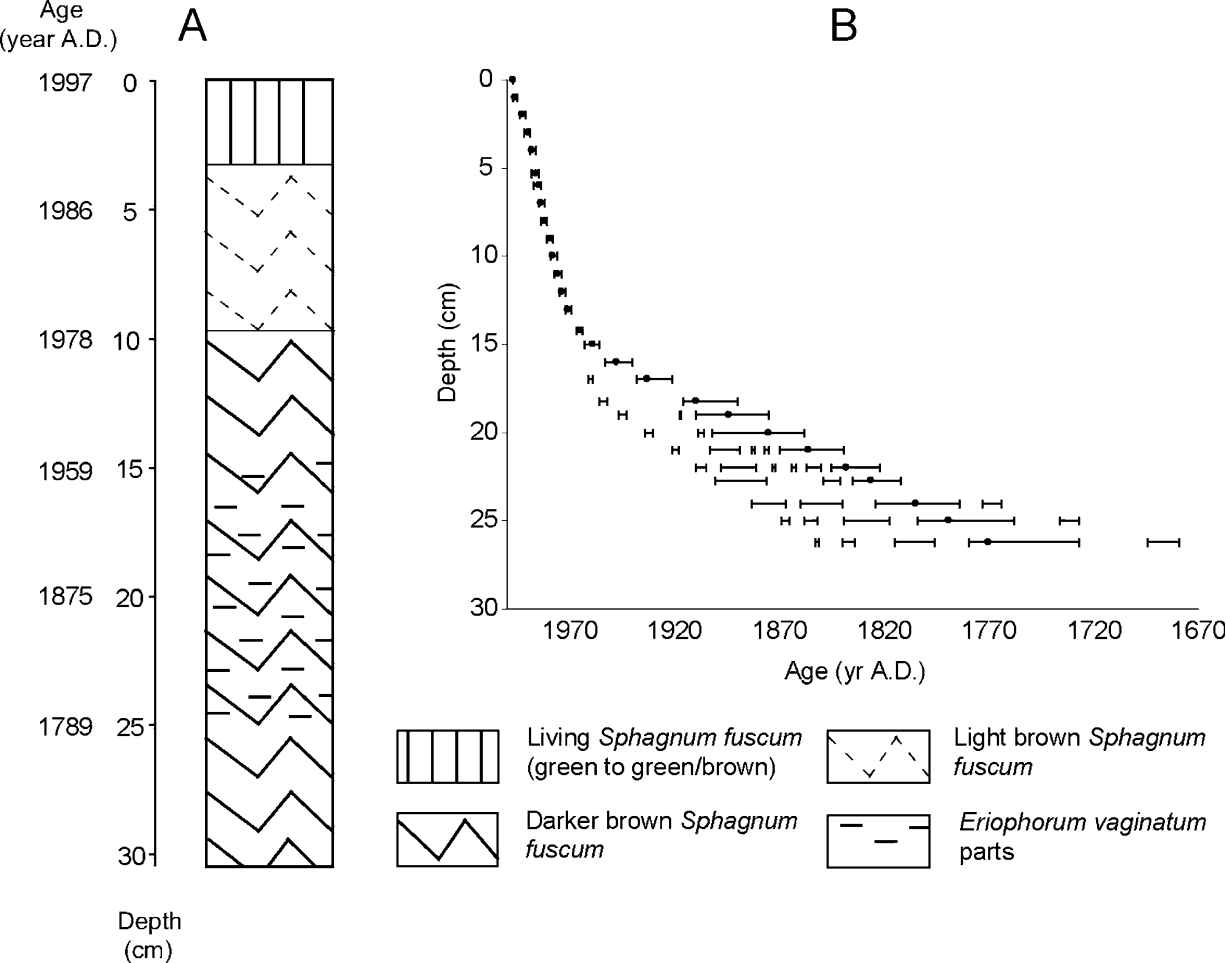
**a** Age, depth and stratigraphy. **b** Age-depth model. *Horizontal bars* represent the 95% confidence intervals of the age-depth model at the selected depths. The best-fit model is represented by *dots*

Our sample site was a small Aapa mire (area 0.75 ha) in northern Finland (69°45.005′ N, 26°59.861′ E, 152 m a.s.l.) near the Kevo Subarctic Research Station. A 60-cm-long peat profile was taken from here in 1997 with a 21-cm-diameter Clymo peat corer. The core, of which the top 30.5 cm have been analysed at high resolution, was taken in a *Sphagnum fuscum* hummock-like string that lacked any other surface vegetation. The core itself consisted mainly of subfossil peat mosses with traces of leaves, grasses, wood and roots (Fig. 1a). Parts of *Eriophorum vaginatum* appear more frequently between 15 and 25 cm with a maximum at 20 cm depth. Because the core itself consists predominantly of *S. fuscum*, a typical hummock species (Hogg 1993), we assume that the sampled section of the peat has always been of the hummock type. The micro-environment at the top of a hummock core is, even within a minerotrophic fen, typical for an ombrotrophic situation. Therefore, we assume that the recorded isotope signature of the sampled peat section also represents an ombrotrophic situation. The regional subarctic climate is characterised by an annual mean temperature of 0°C and a mean July temperature of 13°C. Mean annual precipitation is approximately 435 mm, and the growing season lasts for 110–120 days.

### Laboratory Methods

The core was frozen, cut into contiguous 5-mm slices and air-dried for storage. Depending on the size, sub-samples of the slices were ground by hand, using a pestle and mortar or with a steel ball mill. The powdered material was oven-dried for 24 h at 70°C. The C and N content were determined by dry combustion (CHN elemental analyser, Interscience CE Instruments, analytical precision <0.2%). The δ^13^C and δ^15^N were determined by stable isotope analyses (NA1500 NCS Elemental Analyser—Finnigan Delta plus IRMS, analytical precision <0.1‰).

*S. fuscum* was analysed as a representative plant from the *Sphagnum* carpet, and leaves from *Andromeda polifolia* were measured as a representative of a local vascular plant. The C and N contents were measured on bulk material from all layers and on *S. fuscum* branches (including leaves) from nine layers. The δ^13^C and δ^15^N were determined for 19 layers of bulk material, nine layers of *S. fuscum* branches and *A. polifolia* leaves from eight layers. Bulk samples were selected as pieces of intact core of 1 × 2 × 0.5 cm. Branches (with leaves) of *S. fuscum* and leaves of *A. polifolia* were collected by hand using a stereo binocular microscope.

### Isotope Expression

The results of the C and N isotope analysis are expressed as *δ* (in per mil) according to the following formula:where *R*_sample_ for C is the ^13^C/^12^C ratio of the sample and *R*_standard_ is the ^13^C/^12^C ratio of VPDB. For N, *R*_sample_ represents the ^15^N/^14^N ratio of the sample, and *R*_standard_ is the ^15^N/^14^N ratio of atmospheric N_2_.

### Age

The age-depth model of the profile (Fig. 1b) is based on ten AMS ^14^C dates on *Betula nana* leaves and *Sphagnum* fragments (Goslar et al. 2005). The original age-depth model for this core (Hicks et al. 2004) was determined by hand-drawing the line best fitting the results of calibration of individual ^14^C dates, when plotted on a depth scale in the manner proposed by Goslar et al. (2005). The present age-depth model has been calculated by a more objective method, using the algorithm of free-shape age-depth modelling (Goslar et al. 2009). This algorithm is useful when the age-depth curve is difficult to approximate to a simple mathematical function. The best-fit age-depth model is a reasonable ‘compromise’ between the fit of ^14^C dates to the radiocarbon calibration curve and the general smoothness of the age-depth line. The uncertainty of the free-shape model was assessed with a Monte Carlo Markov chain built from large number of age-depth lines with probabilities dependent on the ‘compromise’ given above.

### Atmospheric CO_2_ and δ^13^C

The historical data for atmospheric δ^13^CO_2_ used in this study were from Francey et al. (1999) and Keeling (2005a). Although atmospheric δ^13^CO_2_ is not homogenous worldwide, a similar decreasing trend is observed globally (Keeling et al. 2005b).

### Atmospheric N Deposition and δ^15^N

No long-term N deposition records that include the unpolluted background deposition levels and their δ^15^N values are available. Ice-core studies that include δ^15^N are rare (e.g. Freyer *et al*. 1996) and lack the necessary high temporal resolution. The Finnish Meteorological Institute has measured wet NO_3_^−^ and NH_4_^+^ deposition at Kevo since 1981 (Kulmala et al. 1998), but dry N deposition has not been measured. The available data from the Kevo station are, in any case, too short to record the initial increase in atmospheric N deposition in the second half of the twentieth century, as seen in data from other Finnish and European measuring stations (Thomas et al. 1988). To estimate the N deposition at Kevo prior to 1981, data for wet N deposition from central Sweden going back to 1955 was used (L. Granat, Department of Meteorology of the Stockholm University, unpublished). The absolute value of deposition in central Sweden was, however, higher than at the Kevo site. We, therefore, used the temporal variation (measured as percentage fluctuations) from the central Swedish data before 1981 to estimate the wet deposition at Kevo before 1981. Measurements of dry deposition from Scandinavia prior to 1980 are extremely scarce or absent. In order to make correlations with historical N deposition data, we used wet N deposition data only. This leads to underestimation of total N deposition, and δ^15^N in wet and dry deposition can differ (Freyer 1991). However, wet deposition contributes more to the total N deposition than dry deposition (Chen et al. 2011; Lawrence et al. 2000), with a dry-to-wet ratio of 0.45:0.19. As wet deposition contributes more to total N deposition, we assume that the main effects presented in this study would not have changed dramatically if dry deposition data had been available. The Kevo combined record of direct and extrapolated data shows a total wet N deposition level of 0.2 kg ha^−1^ year^−1^ in 1955 which slowly rises to a general peak between 1983 and 1993 with values of maximally 0.6 kg ha^−1^ year^−1^ and eventually decreases to 0.4 kg ha^−1^ year^−1^ in 2002 (see Fig. 4).

No data for δ^15^N in precipitation are available for the Kevo region. Bauer et al. (2000) show that although ammonium frequently has a lower δ^15^N than nitrate, δ^15^N in bulk precipitation varies greatly across Europe. Therefore, extrapolation of isotope values in Finland from precipitation nearby, for example in Sweden, is not useful. To study the relation between the isotope signature of N deposition and the isotope signature of the organic matter, we assume that, prior to the onset of anthropogenic N deposition, as in most natural ecosystems, the studied peat bog received its N input mainly from N fixation and to a small degree from atmospheric deposition (Galloway et al. 2004). We therefore assume that the δ^15^N value of pre-anthropogenic N input is close to 0‰ (Bedard-Haughn et al. 2003). As nearly no fractionation occurs when an element is limiting to a reaction, the isotopic value of the N source will be integrated into the sink As deposition increased over time, the enhanced nitrogen availability: (1) allowed for greater biotic discrimination against ^14^N, resulting in lower δ^15^N values (Inglett et al. 2007); (2) at some point became a substantial source of N for plants next to naturally fixed N and (3) generally had a lower δ^15^N than naturally fixed N. Because these three processes are related and enhance each other, the organic matter produced will become increasingly depleted towards the top of the core. Because the N deposition level remains below the saturation level of *Sphagnum*, all extra N (with a δ^15^N value different from 0‰) will be absorbed and incorporated, leading to an altered isotopic value and not a different tissue N concentration or C/N ratio (Lamers et al. 2000). Based on these processes, the combined dataset was used to calculate correlations, first between wet N deposition and bulk peat δ^15^N and, second, between wet N deposition and *S. fuscum* δ^15^N.

In the calculation of the relationship between wet N deposition level and the δ^15^N in bulk peat and *S. fuscum*, two extra values of N deposition for years prior to 1955 were added (1951 for bulk peat and 1946 for *S. fuscum*). These years were assigned to have the estimated wet N deposition value of 1955 (i.e. the oldest historical value). We assume this to be correct or, at worst, a slight overestimation of the level of wet N deposition at the time, which is known to have increased from approximately 1950 onwards.

### Statistical Analysis

An independent samples *t* test (*n* = 20) was conducted to compare the isotope signatures of bulk peat δ^13^C and δ^15^N for pre- and post-anthropogenic periods (i.e. younger and older than 1850 for C and younger and older than 1950 for N). We subsequently analysed whether the vertical gradients in δ^13^C and δ^15^N in this profile are correlated with atmospheric changes in the described parameters. The atmospheric δ^13^CO_2_ for the period 1820–1990 follows a negative exponential curve (Francey et al. 1999). If those values are incorporated into the age-depth model of the peat core, corrected for compaction and decomposition, they appear nearly linear. We therefore studied the relationship between the change in atmospheric δ^13^C over time and the bulk peat, *S. fuscum* and *A. polifolia* δ^13^C with core depth using linear regression analysis. The change in δ^15^N of the peat core is not expected to follow a linear model because the increase in atmospheric N deposition in Europe did not start before the second half of the twentieth century (Pitcairn et al. 1995; Freyer et al. 1996), reached a maximum around 1980 and subsequently decreased. Therefore, the relationship between bulk peat, *S. fuscum* and *A. polifolia* δ^15^N with core depth and the course of δ^15^N over time were analysed with a third-order polynomial using cubic curve estimation combined with an ANOVA. The sample sizes of *S. fuscum* (*n* = 9) and *A. polifolia* (*n* = 8) were rather small. Consequently, the slopes of the models for atmospheric δ^13^CO_2_ and bulk peat δ^13^C were compared according to the method of Zar (1999). To test if decomposition or diagenesis altered the original record (Jones et al. 2010; Sharma et al. 2005), the correlations between bulk peat C/N and bulk peat δ^13^C and δ^15^N were calculated (*n* = 20). All analyses were performed with SPSS 11.0 for Windows.

## Results

**Fig. 2:**
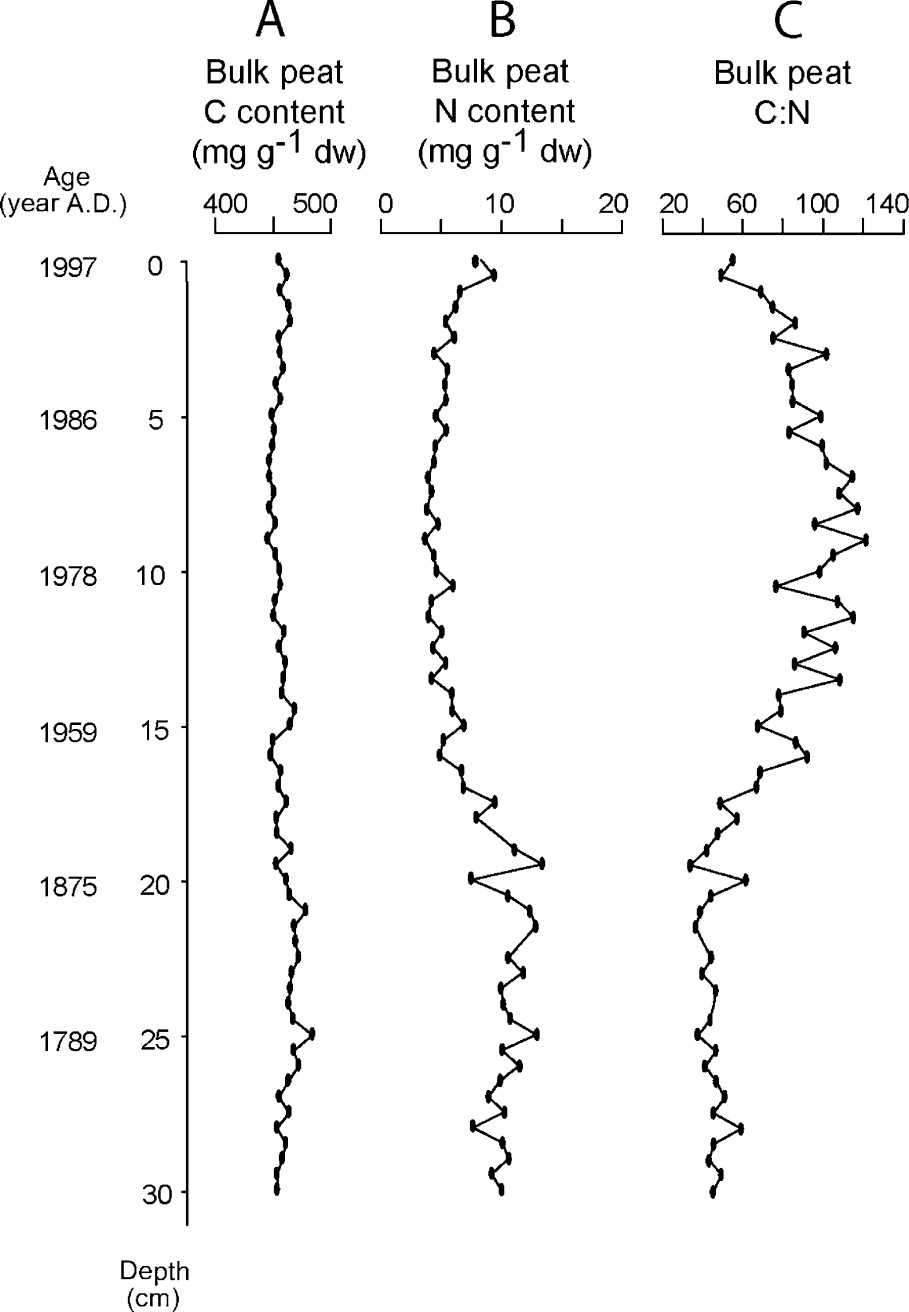
**a** Bulk peat C content (milligrams per gram dw). **b** Bulk peat N content (milligrams per gram dw). **c** Bulk peat C/N with depth (centimetres)

The bulk peat C content is stable around 458 mg g^−1^ dry weight through the entire core (Fig. 2a). The bulk peat N ranges between 4 and 13 mg N g^−1^ (Fig. 2b). It starts just below 10 mg N g^−1^ in the bottom of the core, fluctuates across the next 10 cm upwards, then gradually decreases to stabilize between 15 and 3 cm depth around a value of 4.5 mg N g^−1^. It increases to approximately 8 mg N g^−1^ in the topmost layer. The variations in N content result in the opposite pattern in the bulk peat C/N ratio (Fig. 2c). There was no correlation between C/N and δ^13^C variation in the core (δ^13^C = −0.001 × CN − 26.668, *r* ^*2*^ = 0.0009, *n* = 20, *p* = 0.893), while there was a strong negative correlation between C/N and δ^15^N (δ^15^N = −0.1017 × CN + 1.9787, *r* ^*2*^ = 0.4789, *p* = 0.001). The changes in C and N content of the *S. fuscum* samples along the core (not shown) were similar to those of the bulk peat samples. The C content was stable but slightly lower than that of the bulk peat. The N content was generally higher than that in the bulk samples (10–15 mg g^−1^ dw). The average C/N ratio of *S. fuscum* samples is 27% lower than that of bulk peat samples at the bottom of the core (33 versus 45, respectively).

**Fig. 3:**
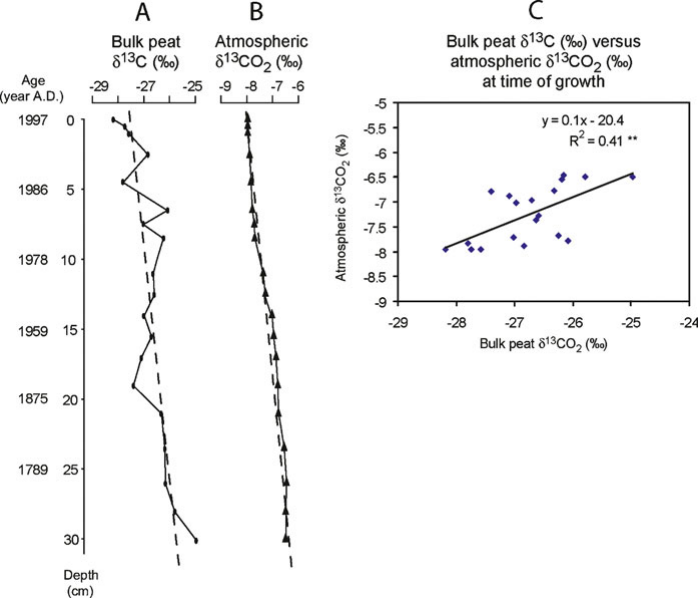
**a** Bulk peat δ^13^C (in per mil) throughout the core. **b** Atmospheric δ^13^CO_2_ (in per mil) for the same period. **c** Bulk peat δ^13^C (in per mil) versus atmospheric δ^13^CO_2_ (in per mil) at time of growth. Data on atmospheric δ^13^CO_2_ adapted from Francey et al. (1999) and Keeling et al. (2005). The standard used for the ^13^C/^12^C is VPDB

The bulk peat δ^13^C values (Fig. 3a) gradually decreased from the bottom to the core top (δ^13^C = 0.06 × depth − 27.5, *r* ^*2*^ = 0.54, *p* < 0.001; Table 1) and fluctuated more rapidly in the more recently formed peat. There was a significant difference in bulk peat δ^13^C signatures of pre-1850 (*M* = −25.78, SD = 0.56) and post-1850 samples (*M* = −26.98, SD = 0.61; *t*_[18]_ = −3.56, *p* = 0.002). The magnitude of the differences in the mean was large (eta squared = 0.41). The overall decreasing shift in bulk peat δ^13^C values is similar to that found for atmospheric δ^13^CO_2_ for the same period (Table 1; Francey et al. 1999). The slopes of both bulk peat δ^13^C and atmospheric δ^13^CO_2_ over the corresponding period (Fig. 3b) are not significantly different from each other. The absolute shift in bulk peat δ^13^C (from 0 to 30 cm) is, however, at least twice as large as the one in atmospheric δ^13^CO_2_. Bulk peat δ^13^C is significantly positively correlated with atmospheric δ^13^CO_2_ at the time of peat formation (δ^13^C = 0.1 atm δ^13^CO_2_  − 20.4, *r* ^*2*^ = 0.41, *p* < 0.01; Fig. 3c).

**Table 1:** Linear regression analyses between the variables given in the first column and core depth

	Model equation	*n*	*r* ^*2*^	*F*	*p*
Atmospheric δ^13^C	$y = 0.058x - 8.017$	19	0.96	406.350	0.000*
Bulk peat δ^13^C	$y = 0.060x - 27.521$	20	0.54	21.309	0.000*
*S. fuscum* δ^13^C	$y = 0.019x - 27.003$	9	0.07	0.539	0.487
*A. polifolia* δ^13^C	$y = 0.049x - 28.132$	8	0.22	1.699	0.240

*p < 0.001

**Fig. 4:**
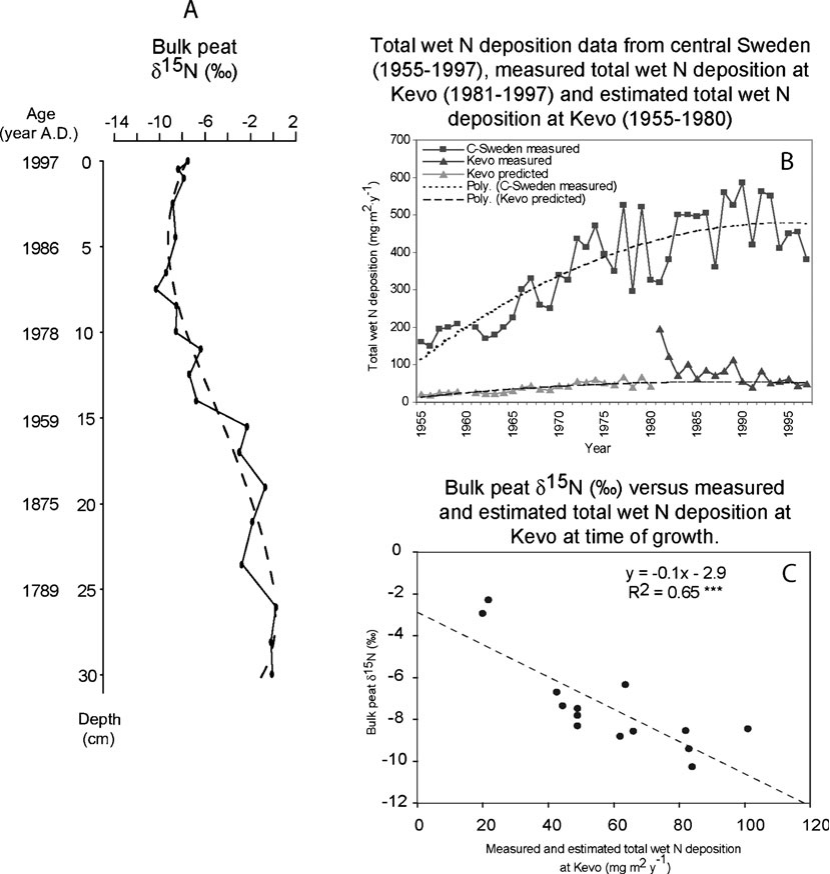
**a** Bulk peat δ^15^N (in per mil) throughout the core. **b** Total wet N deposition data from central Sweden (1955–1997), measured total wet N deposition at Kevo (1981–1997) and estimated total wet N deposition at Kevo (1955–1980). **c** Bulk peat δ^15^N (in per mil) versus measured and estimated total wet N deposition at Kevo (in milligrams per square metre per year). Data on total wet N deposition at Kevo were measured from 1981 to 1997 (Kulmala *et al*. 1998). Estimates for the period between 1955 and 1981 were made using temporal variation in a central Swedish dataset provided by Dr. Lennart Granat from the Department of Meteorology, Stockholm University. The standard used is the ^15^N/^14^N of atmospheric N_2_

Bulk peat δ^15^N values (Fig. 4a) are around 0‰ in the bottom part of the core. They gradually decrease upward and finally stabilize in the top part at a value of around −7.5‰. The stratigraphic change in bulk peat δ^15^N (*r* ^*2*^ = 0.91, *n* = 20, *F* = 0.00; Table 2) is very strong. There was a significant difference in bulk peat δ^15^N signatures of pre-1950 (*M* = −1.18, SD = 1.32) and post-1950 samples (*M* = −7.73, SD = 1.94; *t*_[18]_ = −7.94, *p* < 0.001). The magnitude of the differences in the mean was large (eta squared = 0.78). Bulk peat δ^15^N is strongly negatively correlated with the available historical measured and estimated wet N deposition values at Kevo (δ^15^N = −0.1 × estimated total wet N deposition at Kevo − 2.9, *r* ^*2*^ = 0.65, *n* = 14, *p* = 0.001; Fig. 4b, c).

**Table 2:** Third-order polynomial regression analyses between the variables given in the first column and core depth

	Model equation	*n*	*r* ^*2*^	*F*	*p*
Bulk peat δ^15^N	$y = - 0.0019x3 + 0.088x2 - 0.7173x - 7.6251$	20	0.91	56.117	0.000**
*S. fuscum* δ^15^N	$y = - 0.0006x3 + 0.015x2 + 0.4848x - 12.9486$	9	0.88	11.891	0.010*
*A. polifolia* δ^15^N	$y = - 0.0013x3 + 0.048x2 - 0.4051x - 6.3518$	8	0.16	0.975	0.369

*p ≤ 0.01; **p < 0.001

**Fig. 5:**
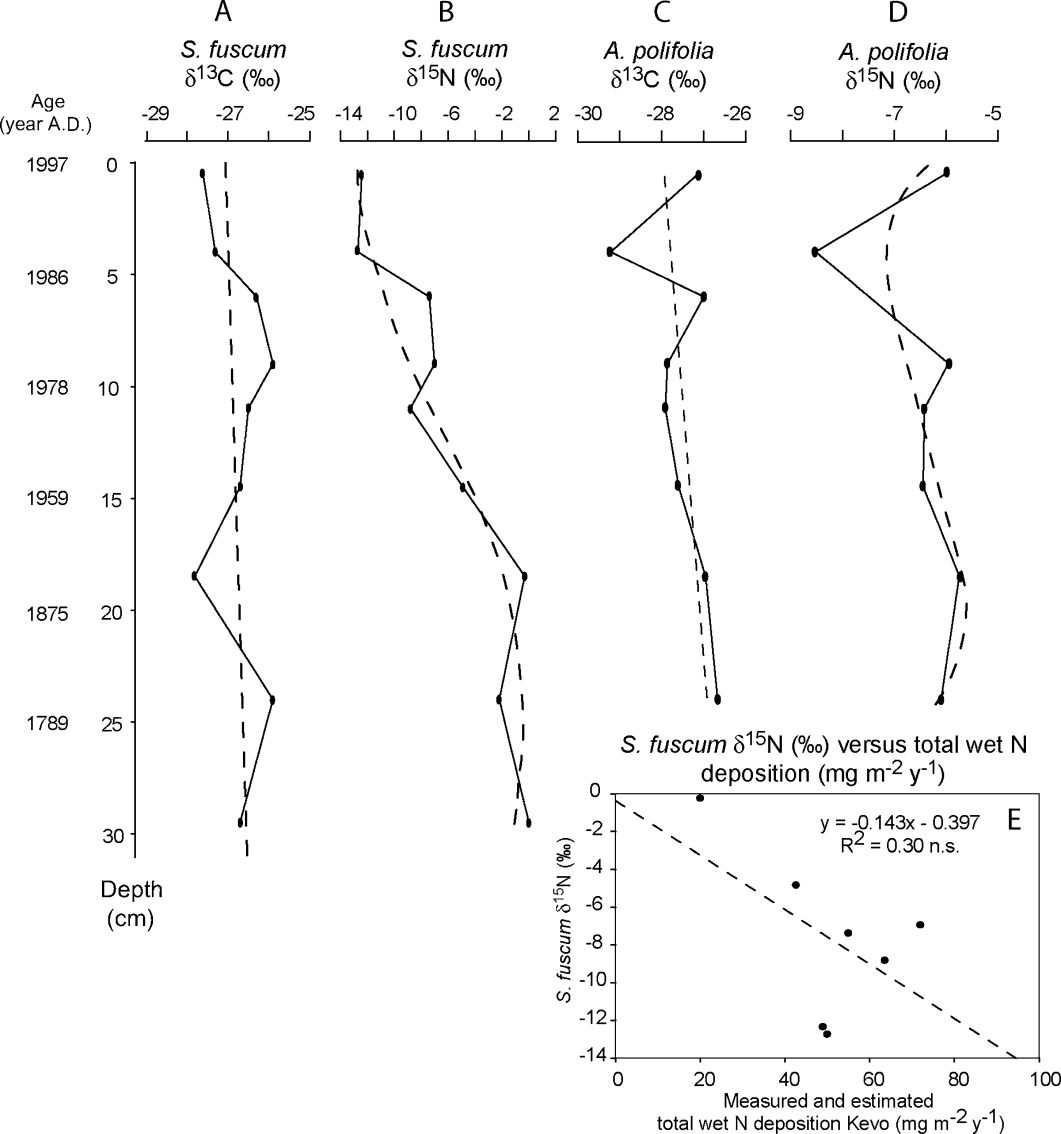
**a** *S. fuscum* δ^13^C (in per mil) throughout the core. **b** *S. fuscum* δ^15^N (in per mil) throughout the core. **c** *A. polifolia* δ^13^C (in per mil) throughout the core. **d** *A. polifolia* δ^15^N (in per mil) throughout the core. **e** *S. fuscum* δ^15^N (in per mil) versus total wet N deposition (milligrams per square metre per year)

The δ^13^C of *S. fuscum* did not change significantly with depth (*r* ^*2*^ = 0.07, *n* = 9, *p* = 0.487; Fig. 5a; Table 1), nor was there any significant relationship between *S. fuscum* δ^13^C and atmospheric δ^13^CO_2_ (*r* ^*2*^ = 0.06, *n* = 9, *p* = 0.531). The decrease in *S. fuscum* δ^15^N with decreasing depth (Fig. 5b) is similar to that found for bulk peat (Fig. 4a). The correlation between *S. fuscum* δ^15^N and depth is high (*r* ^*2*^ = 0.88, *n* = 9, *p* = 0.01; Table 2). There was, however, no significant relationship between *S. fuscum* δ^15^N and the measured and predicted total wet N deposition at Kevo (*r* ^*2*^ = 0.30, *n* = 7, *p* = 0.201; Fig. 5e).

The *A. polifolia* leaf δ^13^C values (Fig. 5c) showed no statistically significant relationship with depth (*r* ^*2*^ = 0.22, *n* = 8, *p* = 0.24; Table 1) or atmospheric δ^13^CO_2_ at the time of growth (*r* ^*2*^ = 0.19, *n* = 8, *p* = 0.277). The correlation between *A. polifolia* δ^15^N and depth (Fig. 5d) was also not significant (*r* ^*2*^ = 0.16, *n* = 8, *F* = 0.37; Table 2). Moreover, there was no relationship between *A. polifolia* δ^15^N and total wet N deposition at Kevo (*r* ^*2*^ = 0.01, *n* = 6, *p* = 0.831). Most values scattered around −6‰, except for the sample at 4 cm depth, which had a value of −8.56‰.

## Discussion

### Bulk Peat and *S. fuscum* C and N

The observed patterns in N content and C/N ratio of bulk peat and *S. fuscum* samples from the core are similar to those found in other studies (Asada et al. 2005; Benner et al. 1991; Kuhry and Vitt 1996; Malmer and Wallen 2004; Moore et al. 2004). Although *Sphagnum* is known for its highly resistant litter quality and can create an adverse micro-environment (van Breemen 1995; Verhoeven and Liefveld 1997; Verhoeven and Toth 1995), it is subject to decomposition (Asada et al. 2005; Blodau 2002; Clymo 1984; Limpens et al. 2008; Limpens and Berendse 2003b). Phases of aerobic decomposition of the younger peat in the acrotelm (0 to ±10 cm) and immobilisation (10–20 cm) and stability (20–30.5 cm) in the catotelm are recognizable (Kuhry and Vitt 1996). The generally higher N content of *S. fuscum* branches compared to bulk peat may be caused by a difference in C/N ratio of stems and branches, as was found for δ^13^C in *Sphagnum* (Loader et al. 2007).

To test if the chemical composition of the organic matter, specifically if the ratio of degradable sugar-like compounds relative to refractory lignin-like compounds, changed from the top of the core to the bottom, two pyrolysis gas chromatography–mass spectrometry analysis were additionally performed (Online Resource). Single samples of the top and bottom core material were analysed. The results indicate that the relative amount of easily degradable sugar-like compounds decreases with depth and that the relative amount of lignin- and lipid-like compounds increases, which indicates compound-selective decomposition (Benner et al. 1987; Orem and Hatcher 1987; Smith and Jacobson 1976; Spiker and Hatcher 1987; Stout et al. 1988; Swift et al. 1979; Wedin et al. 1995).

### Relationship Between the Dilution of δ^13^C in Atmospheric CO_2_ and Bulk Peat, *S. fuscum* and *A. polifolia*

The bulk peat δ^13^C values decreased from the bottom to the top of the core. As hypothesised, the bulk peat δ^13^C of pre-1850 samples was significantly higher than that from post-anthropogenic periods. In agreement with Loisel et al. (2010), this shift is significantly positively correlated with atmospheric δ^13^CO_2_ at the time of growth. The absolute shift in bulk peat δ^13^C (from 0 to 30.5 cm) is, however, at least twice as large as the one in atmospheric δ^13^CO_2_.

Although the C/N ratio and PGCMS analysis indicate decomposition, there was no correlation between C/N ratio and bulk peat δ^13^C. This may indicate that decomposition has not significantly altered the original atmospheric δ^13^CO_2_ record (Sharma et al. 2005) and points towards decomposition with a preferential loss of N (versus C) (Asada et al. 2005).

A small change in the species composition has occurred as some *E. vaginatum* remains were found between 15 and 25 cm and could potentially have biased the N-isotope record. While *Sphagnum* lacks a root system, *E. vaginatum* can penetrate the peat matrix and use recycled N from the ground. However, the trend in bulk peat δ^15^N is similar to that measured in *S. fuscum* leaves. Consequently, the shift in bulk peat δ^15^N (or δ^13^C) is not related to the occurrence of the *E. vaginatum* remains.

The observed change in bulk peat δ^13^C in this study (−3.2‰ from 1820 to 1997) is in agreement not only with several other studies for the same period (Ficken et al. 1998; Macko et al. 1991; Novak et al. 1999) but also with other studies covering a time interval before 1850 (Hendon et al. 2001). This reinforces the idea that processes other than increasing atmospheric CO_2_ are influencing peat isotope chemistry. Compound-specific decomposition would result in a larger fraction of recalcitrant substrates and decreased δ^13^C with depth and/or decomposition (Benner et al. 1987; Dorrepaal et al. 2009; Loisel et al. 2010; Melillo et al. 1989; Menot and Burns 2001; Wedin et al. 1995). Instead, the bulk peat δ^13^C in our core increases with decomposition stage. Kinetic fractionation against ^13^C during decomposition and respiration (Agren et al. 1996) may have caused an increase in δ^13^C in the residual material. However, this process is not fully understood (Bostrom et al. 2007; Ekblad et al. 2002; Fernandez and Cadisch 2003). The observed change in δ^13^C is in its direction and magnitude also consistent with a gradual shift in the relative contribution of microbial, as opposed to plant material (Ehleringer et al. 2000; Taylor et al. 2003; Wallander et al. 2004). Another factor that has to be taken into consideration is the air directly surrounding *Sphagnum*. It may be affected by both plant and microbial respiration and, combined with decomposition of the underlying peat layers, may not be representative for modern atmospheric CO_2_ (Price et al. 1997; Smolders et al. 2001). The observed ^13^C-depletion in all materials in the upper layers is consistent with re-fixation (Raghoebarsing et al. 2005; Turetsky and Wieder 1999). Because *A. polifolia* grows as a low-creeping plant, we were unable to use it as an indicator of free atmospheric conditions.

Despite the overall trend in bulk δ^13^C, there are no correlations between either depth or atmospheric δ^13^CO_2_ and both *S. fuscum* and *A. polifolia* δ^13^C. This could be, however, also the result of a smaller sample size for the species specific δ^13^C (*n* = 9) than for bulk peat δ^13^C (*n* = 20), an example of statistical type II error. *S. fuscum* δ^13^C should be more sensitive to changes in δ^13^CO_2_ than bulk peat because decomposition processes are likely to be more intense in the peat matrix than on individual *Sphagnum* plant parts. Although we did find signs of kinetic decomposition in bulk peat, we did not find statistically significant evidence of decomposition for *S. fuscum* δ^13^C. Therefore, decomposition may well act as a driving force for the shift in δ^13^C, instead of merely enhancing or overlapping the signature of decreasing atmospheric δ^13^CO_2_.

### Relationship Between Atmospheric N Deposition and Bulk Peat, *S. fuscum* and *A. polifolia* δ^15^N

As hypothesised, the pre-1950 bulk peat δ^15^N values were significantly higher than those from post-anthropogenic periods. Furthermore, our results indicate that 65% of the variance in bulk peat δ^15^N in the core can be explained by historical (measured and estimated) wet N deposition values.

The C/N ratio shows a preferential loss of N (versus C) during aerobic decomposition in the upper 10 to 12 cm (Lehmann et al. 2003). After that, the C/N decreases because of methanogenesis (Kuhry and Vitt 1996) and stabilizes as nitrogen becomes immobilized in the peat. In contrast to the absent correlation between the C/N ratio and δ^13^C, there is a relatively strong relationship between C/N and δ^15^N. This indicates that decomposition or diagenesis has significantly altered the original record. Asada et al. (2005) already showed that ^15^N enrichment during the first years can be used as a proxy for decomposition. Furthermore, the changes in C/N and δ^15^N found in their Canadian peat core are very similar to those found in our Finland core.

There are still several other processes that can alter the bulk peat the δ^15^N. We have already ruled out a change in the species composition (see previous section). Kinetic fractionation resulting from a number of processes, including decomposition, nitrification, denitrification and microbial incorporation of N, may explain part of the enrichment in the deeper layers (Bedard-Haughn et al. 2003; Francez et al. 2000; Nadelhoffer and Fry 1994; Turner et al. 1983). The observed enrichment in the present study is comparable to that found in soil profiles (Hobbie and Ouimette 2009). Moreover, specifically in peat, transport of N in the upper living part of the apex may also play a role. Here numerous processes occur, which can impact the δ^15^N (and δ^13^C) of the organic material. Leaching, by contrast, is one of the few processes that favours the heavier ^15^N isotope due do the influence of gravitation, although the size of this effect is unknown (Bedard-Haughn et al. 2003) and may have taken place in our study. In the end, however, it is clear that these other processes occur simultaneously with the change in N deposition and the shift in δ^15^N and may enhance it.

The significant and strong correlation between *S. fuscum* δ^15^N and depth is most likely caused by preferential loss of N during aerobic decomposition as there was no significant correlation between *S. fuscum* δ^15^N and the measured and predicted total wet N deposition at Kevo. As with δ^13^C, the absence of significance in the similar trending *S. fuscum* signal may be due to a type II error on the low number of samples analysed. Kinetic decomposition can enhance or overlap the correlation between *S. fuscum* δ^15^N and the measured and predicted total wet N deposition. Although the absolute change in the core may be similar to changes in herbarium vascular and bryophyte samples over time (Stewart et al. 2002), the changes in the core may also be up to three times larger (Penuelas and Filella 2001). When comparing the shift in our core to δ^15^N shifts in other soil and peat profiles (Bedard-Haughn et al. 2003; Gebauer et al. 1994; Kohzu et al. 2003; Nadelhoffer and Fry 1994), there is a general pattern of enrichment with depth independent of sample age. However, the absolute change in the core of Kohzu et al. (2003) is much smaller than in our core.

Surprisingly, circumstantial evidence from the *A. polifolia* leaves does not point to a major role for kinetic discrimination in soil processes. As the level of wet N deposition remains below the N saturation point of *Sphagnum*, the wet N deposition is probably not available for uptake by *A. polifolia*, as its roots are located below the living *Sphagnum* carpet. Nordbakken et al. (2003) also found that the filtering capacity of *Sphagnum* mosses prevented uptake of N treatment by vascular plants that rooted below a living *Sphagnum* layer. The value of *A. polifolia* δ^15^N itself may be indicative of a mycorrhizal association, although the results of studies of this association on δ^15^N seem to be highly variable (Spriggs et al. 2003; Zimmer et al. 2007). Although *A. polifolia* is assumed to be less refractory than *Sphagnum*, the general value of *A. polifolia* δ^15^N throughout the core, at around −6‰, remains relatively stable. It therefore appears that kinetic fractionation during, for example, decomposition is not of major influence.

Although the N deposition has changed over time at Kevo, it has remained very low. The absolute values range from 20 mg m^−2^ year^−1^ in 1955 to 194 mg m^−2^ year^−1^ in 1981 to 49 mg m^−2^ year^−1^ in 1997. If this is compared to the amount of natural N fixation, which can be around 200 mg m^−2^ year^−1^ in peat (Cleveland et al. 1999; Deluca et al. 2002), it is clear that the contribution of N deposition to total N availability may only have been approximately 9% in 1955 (200 + 20 mg), 49% in 1981 (200 + 194 mg) and 20% in 1997 (200 + 49 mg). Therefore, we have to take into account a dilution effect on measures of δ^15^N. However, because natural fixation has a value near 0‰, more negative depositional values will still be apparent.

## Conclusions and Future Work

We conclude that bulk peat stable carbon and nitrogen isotope ratios may not only reflect the history of increased atmospheric carbon dioxide and nitrogen deposition in the past 50–150 years but probably also reflect the effects of early stage kinetic fractionation during diagenesis with a preferential loss of N over C. δ^13^C in bulk peat is a moderate environmental proxy for the dilution of atmospheric ^13^CO_2_. Although the indicative value of bulk peat δ^15^N as an environmental proxy for total wet N deposition is significant and relatively high, the correlation with bulk peat C/N is also significant and strong. Our study shows that the depleted atmospheric ^13^CO_2_ and depleted N deposition may very well contribute to the observed shift in δ^13^C and δ^15^N, but their effects are enhanced or superimposed by kinetic fractionation during decomposition. Nevertheless, our study shows that both need to be taken into account in interpreting palaeodata in general.

To improve the reliability of these elements as biological proxies, additional research is necessary: (1) The data on atmospheric N deposition should also include detailed isotopic analysis of both wet and dry deposition. Because these were not available in the present study, we could only relate the isotope chemistry of the core to the amount of wet N deposition and (2) δ^15^N needs to be measured in more peat cores, ideally from sites with different rates of decomposition at a high resolution and in combination with age-depth modelling. It may be possible to correct for kinetic fractionation and reveal the extent of the deposition effect.

Our results show that the Kevo peat can be used as natural archives reflecting environmental changes. It clearly indicates a change of the carbon isotope ratio on a global scale, as a result of the release of large amounts of CO_2_ originating from the combustion of fossil fuels along with an increasing land use change. Variations in δ^15^N, however, are rather a result of natural fractionation processes, masking anthropogenic changes in the δ^15^N of N deposition.

## Electronic Supplementary Material

Below is the link to the electronic supplementary material.

Online Resource The ratio of degradable sugar-like compounds relative to refractory lignin-like compounds in a top and bottom single sample pyrolysis gas chromatography–mass spectrometry measurement of a 30-cm-long peat core from Kevo, Finland (PDF 390 kb)
