# Supplementary material for: Stable Carbon and Nitrogen Isotopes in a Peat Profile Are Influenced by Early Stage Diagenesis and Changes in Atmospheric CO2 and N Deposition
Source: Water Air Soil Pollut. 2012 Jan 25;223(5):2007–22. doi: 10.1007/s11270-011-1001-8 (PMC3359457; doi:10.1007/s11270-011-1001-8)

Stable carbon and nitrogen isotopes in a peat profile are influenced by early stage diagenesis, and changes in atmospheric CO_2 and N deposition.

A.J. Esmeijer-Liu[1 2], W.M. Kürschner[1], A.F. Lotter[1], J.T.A. Verhoeven[2], T. Goslar[3 4].

1 Utrecht University, Institute of Environmental Biology, Palaeoecology, Budapestlaan 4, 3584 CD Utrecht, the Netherlands
2 Utrecht University, Institute of Environmental Biology, Ecology and Biodiversity, Padualaan 8, 3584 CA, Utrecht, the Netherlands
3 A. Mickiewicz University , Faculty of Physics, Umultowska 85, 61-614 Poznan, Poland
4 Poznań Radiocarbon Laboratory, ul. Rubież 46, 61-612 Poznań, Poland

Water, Air and Soil Pollution

Corresponding author:
A.J. Esmeijer-Liu, tel: +31 30 2536856, fax:+31 30 2518366 , email: A.J.Liu@uu.nl

Pyrolisis - Gas chromatography–mass spectrometry (py-GC/MS)

GC analyses were performed using a Hewlett–Packard 6890 series gas chromatograph equipped with a CP-sil 5CB silica column (50 m×0.32 mm, film thickness 0.12 µm). The oven temperature was programmed from 70 to 130 °C at 20 °C min^{-1} and from 130 °C to 320 °C (isothermal for 20 min) at 4 °C min^{-1}. Compounds were detected using a flame ionisation detector at 325 °C. Helium was used as carrier gas.

Gas chromatography–mass spectrometry analyses were performed using a Hewlett–Packard 5890 series II gas chromatograph connected to a Fisons instruments VG platform II mass spectrometer operating at 70 eV, scanning the range *m*/*z* 50–650 with a cycling time of 0.65 s. The capillary column and temperature programme were as described for the GC analyses.

The py-GC-MS traces (Fig. 1) give an overview of the peaks measured by py-GC-MS. Identification was performed using Smeerdijk and Boon (1987). Not all peaks could be identified. The largest peak is phenol. The traces of both samples were compared relative to the largest peak (14.33 and 14.20). After that, a comparison was made between peaks indicative for polysaccharides and lignin/lignin-like compounds.

The two circled groups A and B represent the common trend that is given by the whole picture. In group A there are 4 peaks of interest that are present in both graphs but in different heights (relative quantities). They are indicated with arrows. The first two indicate sugar-like compounds, the last two indicate lignin-like compounds. It is very clear that in the older material there is relatively much more of the lignin-like compounds than in the younger, which is consistent with some degree of decomposition. In group B it can be seen that there are many more and higher peaks in the older material than in the young, and most of these peaks stand for lignin- and lipid-like compounds. This is again consistent with decomposition. Because this analysis does not give exact quantities it is not possible to calculate the quantities of each fraction (sugars, cellulose, lignin, lipids) is present. It is however clear that there is some degree of decomposition down the core.

Figure 1: The results of the py-GC-MS of the Kevo core. Upper graph are the results from the top of the core, lower graph are those from the bottom.

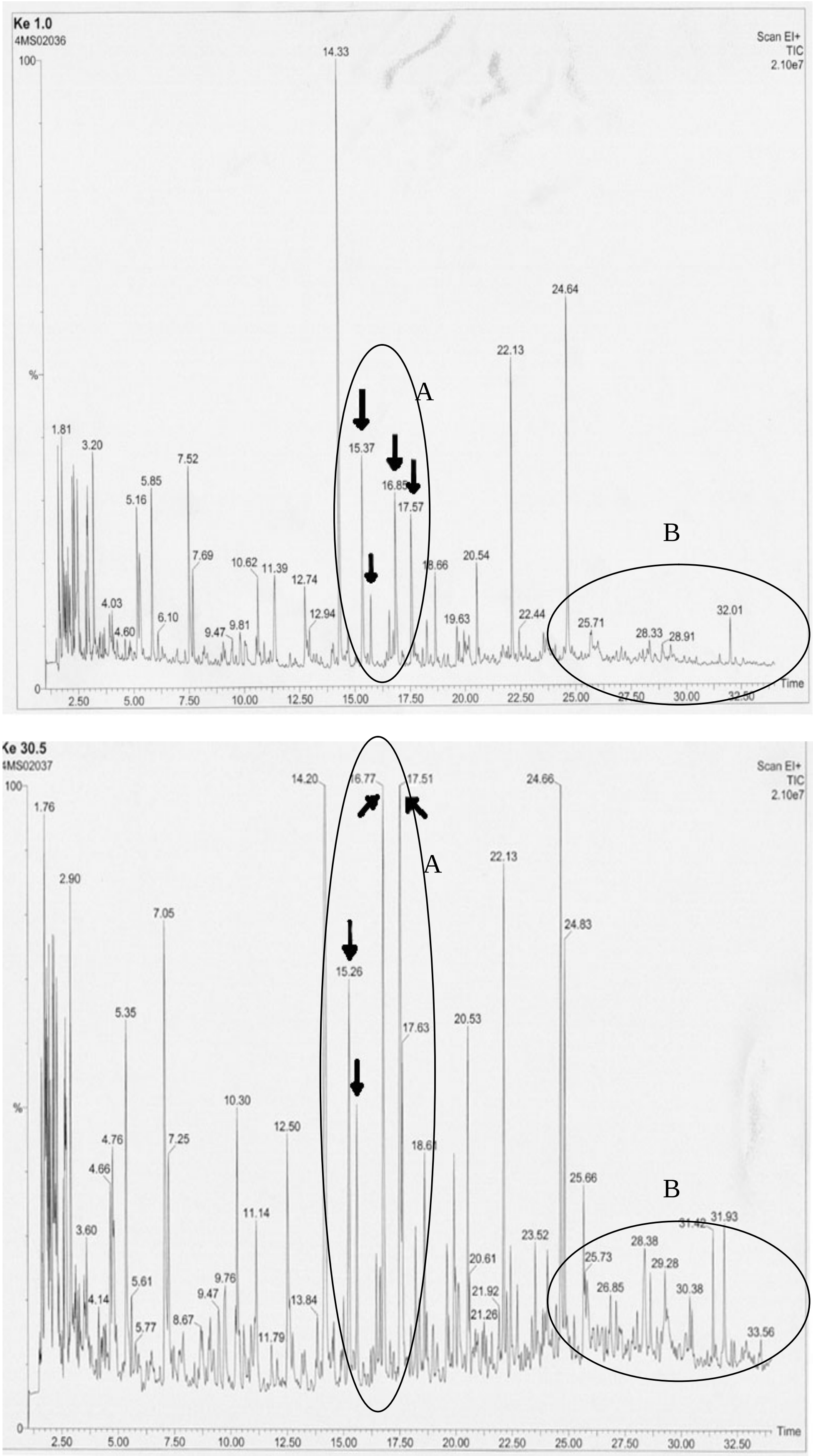

Supplement: Supplementary file 1 — The ratio of degradable sugar-like compounds relative to refractory lignin-like compounds in a top and bottom single sample pyrolysis gas chromatography–mass spectrometry measurement of a 30-cm-long peat core from Kevo, Finland (PDF 390 kb) [file 11270_2011_1001_MOESM1_ESM.pdf]
